# The Internal Architecture of Leukocyte Lipid Body Organelles Captured by Three-Dimensional Electron Microscopy Tomography

**DOI:** 10.1371/journal.pone.0059578

**Published:** 2013-03-26

**Authors:** Rossana C. N. Melo, Guillherme F. Paganoti, Ann M. Dvorak, Peter F. Weller

**Affiliations:** 1 Laboratory of Cellular Biology, Department of Biology, Federal University of Juiz de Fora, UFJF, Juiz de Fora, MG, Brazil; 2 Department of Medicine, Beth Israel Deaconess Medical Center, Harvard Medical School, Boston, Massachusetts, United States of America; 3 Department of Pathology, Beth Israel Deaconess Medical Center, Harvard Medical School, Boston, Massachusetts, United States of America; Fundação Oswaldo Cruz, Brazil

## Abstract

Lipid bodies (LBs), also known as lipid droplets, are complex organelles of all eukaryotic cells linked to a variety of biological functions as well as to the development of human diseases. In cells from the immune system, such as eosinophils, neutrophils and macrophages, LBs are rapidly formed in the cytoplasm in response to inflammatory and infectious diseases and are sites of synthesis of eicosanoid lipid mediators. However, little is known about the structural organization of these organelles. It is unclear whether leukocyte LBs contain a hydrophobic core of neutral lipids as found in lipid droplets from adipocytes and how diverse proteins, including enzymes involved in eicosanoid formation, incorporate into LBs. Here, leukocyte LB ultrastructure was studied in detail by conventional transmission electron microscopy (TEM), immunogold EM and electron tomography. By careful analysis of the two-dimensional ultrastructure of LBs from human blood eosinophils under different conditions, we identified membranous structures within LBs in both resting and activated cells. Cyclooxygenase, a membrane inserted protein that catalyzes the first step in prostaglandin synthesis, was localized throughout the internum of LBs. We used fully automated dual-axis electron tomography to study the three-dimensional architecture of LBs in high resolution. By tracking 4 nm-thick serial digital sections we found that leukocyte LBs enclose an intricate system of membranes within their “cores”. After computational reconstruction, we showed that these membranes are organized as a network of tubules which resemble the endoplasmic reticulum (ER). Our findings explain how membrane-bound proteins interact and are spatially arranged within LB “cores” and support a model for LB formation by incorporating cytoplasmic membranes of the ER, instead of the conventional view that LBs emerge from the ER leaflets. This is important to understand the functional capabilities of leukocyte LBs in health and during diverse diseases in which these organelles are functionally involved.

## Introduction

Intracellular inclusions containing lipids are widely present in eukaryotic cells (from protozoa to mammals) and bacteria. In many cells, including adipocytes, steroidogenic cells and hepatocytes, these inclusions, termed lipid droplets (LDs), are extensively recognized for their roles in neutral lipid storage and metabolism (reviewed in [1]–[2]). LDs in these cells consist of a central repository of neutral lipids, including triglycerides, surrounded by a phospholipid monolayer. Proteins that interact with LDs do so only at the peripheral phospholipid monolayer [3]. While this structural organization of LDs with a solely circumferential phospholipid monolayer underlies the roles of LDs as dynamic organelles pertinent to neutral lipid metabolism, whether all lipid inclusions, including those in leukocytes, have an identical structure has not been ascertained.

In leukocytes, lipid inclusions, often called lipid bodies (LBs), are also dynamic organelles with functions including serving as niduses for synthesis of arachidonic acid-derived inflammatory lipid mediators (eicosanoids) [4]–[8]. LBs are therefore critical to the functional capabilities of cells from the immune system, such as eosinophils, neutrophils and macrophages. In these cells, LBs are rapidly formed in response to a range of inflammatory diseases and are structural markers of inflammatory cells in innate immunity (reviewed in [9]).

With the recognition that leukocyte LBs are sites for the regulated biosynthesis of inflammatory lipid mediators such as prostaglandins and leukotrienes [4]–[7], varied membrane inserting proteins have been associated with LBs. For example, proteomic analyses revealed a detailed list of proteins in lipid bodies, including several membrane-spanning proteins, such as vesicle-associated membrane protein 4 (VAMP4), with predicted membrane insertion domains within these organelles [10]–[11]. ImmunoEM has also shown that caveolin-1 and other membrane proteins are localized inside LBs [4], [12]–[13]. Such findings raise intriguing questions: 1) how are transmembrane proteins localized within LBs? and 2) is the internal core of LBs akin to the hydrophobic core of adipocyte LDs?

Some of the difficulties in the study of LBs originate from their unique architecture. In contrast to vesicles and membranous organelles that have an aqueous content surrounded by a phospholipid bilayer membrane, LBs and LDs are encircled just by a monolayer of phospholipids [14]. LBs, therefore, lack a true delimiting unit membrane structure. In addition, it is not easy to preserve LBs. The lipid content is extracted by drying or fixation and staining with alcohol-based reagents, and the use of fluorescent lipophilic dyes to detect lipid bodies has some limitations [15]. A new generation of microscopic techniques, such as coherent anti-Stokes Raman scattering (CARS) microscopy [16], including multiplex CARS microscopy [17] and third-harmonic generation (THG) microscopy [18], is helpful in the study of LBs, but the resolution of these techniques does not enable the detailed observation of LB substructure.

By conventional transmission electron microscopy (TEM), LBs generally appear as homogeneous organelles. However, the use of freeze-fracture [12], water soluble embedding media [19] or even a comprehensive examination of thin sections prepared by conventional TEM [10] has pointed out that LBs in different cell types may have a complex internal organization and are not solely a mass of lipid esters.

An emerging application of automated electron tomography renders feasible the observation of the three-dimensional (3D) architecture of cellular components [20]. Studies using electron tomography have been redefining our understanding of the organization of several organelles and membrane systems, leading to many new insights into the functional organization of varied cells [21]–[25].

Given the broader functional roles for LBs in cells from the immune system, here we used electron tomography as well as conventional TEM and immunogold EM to study structural aspects of LBs in human eosinophils, cells involved in a diversity of allergic and inflammatory diseases (reviewed in [26]–[27]). We provide evidence for the presence of membranes within LBs and demonstrate, after 3D-reconstruction and modeling, that the LB membranous internal structures are organized as a network of tubules resembling the endoplasmic reticulum (ER). The membranous system observed within LBs explains how membrane-bound proteins are localized within LB “cores” and supports a model for LB formation by incorporating cytoplasmic membranes of the ER, instead of the conventional view that LBs emerge from the ER leaflets. The novel identification of organized internal membranes within LBs provides a new concept of these organelles, differentiating leukocyte LBs in structure and function from adipocyte LDs. These findings bring a new focus on LBs and help to understand the functional capabilities of these organelles.

## Results

### Two-dimensional Ultrastructure of LBs within Human Eosinophils

**Figure 1:**
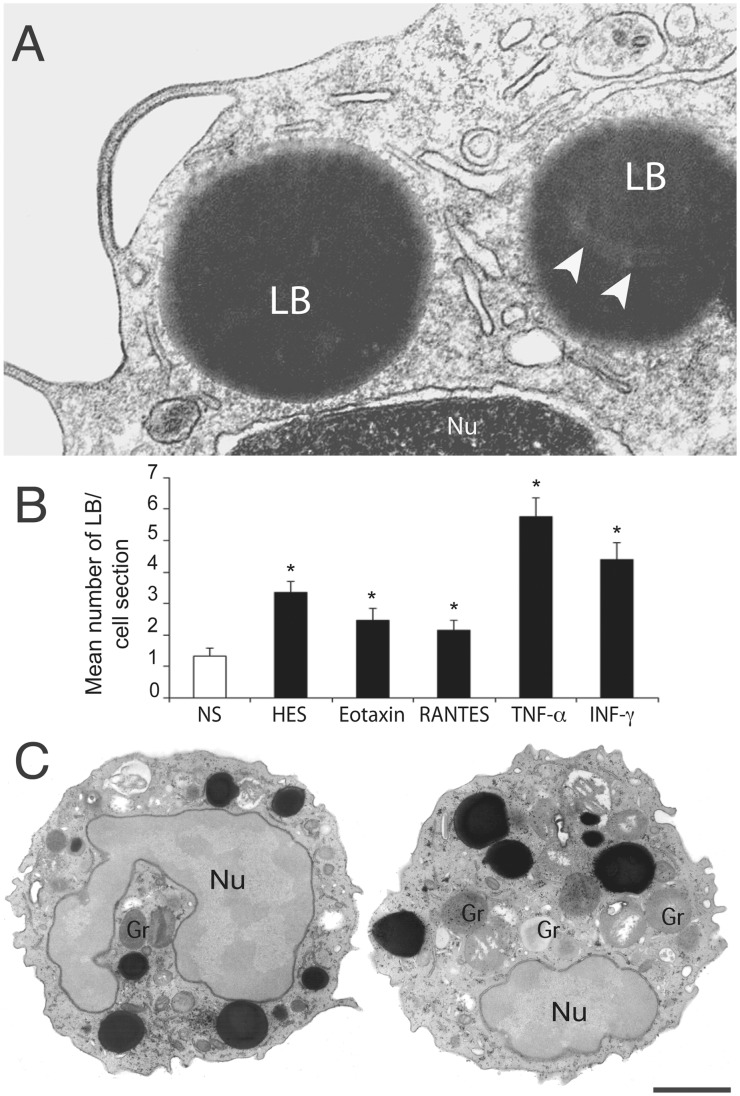
Lipid bodies (LBs), cytoplasmic organelles not delimited by a bilayer membrane, are rapidly formed in response to leukocyte activation. (**A**) Electron micrograph of a conventional thin section (∼ 80 nm thickness) shows typical LBs in the cytoplasm of a resting human eosinophil as round, electron-dense organelles. Variation in the LB density can be observed in the core of one LB (arrowheads). (**B**) Stimulation of eosinophils with inflammatory cytokines induces significant formation (* *P≤0.05*) of LBs in the cytoplasm compared to unstimulated cells. Naturally activated eosinophils from hypereosinophilic patients also show increased numbers of LBs. (**C**) In activated eosinophils from tumor necrosis factor alpha (TNF-α)-stimulated eosinophils, a large number of LBs with different sizes are seen in the cytoplasm as highly contrasted organelles while nucleus and other organelles are imaged with less contrast. Cells were isolated from the peripheral blood of healthy or hypereosinophilic donors, stimulated or not with eotaxin-1/CCL11, RANTES/CCL5, TNF–α or interferon gamma (INF-γ) as in material and methods and processed for transmission electron microscopy (TEM) using conventional (A) or reduced osmium (B). Gr, specific granule; Nu, nucleus. Scale bar, 500 nm (A); 900 nm (C).

Eosinophil LBs are highly osmiophilic organelles and the use of osmium tetroxide is critical to fix and therefore to visualize them by TEM. Our protocol for optimal preservation of purified eosinophils and identification of cytoplasmic LBs includes prompt aldehyde fixation while the cells are still in suspension followed by agar embedding for accurate sample handling and adequate osmium post-fixation in aqueous medium [10], [13]. Thus, eosinophil LBs generally appear as roughly round and very electron-dense organelles in TEM images and the other organelles are well preserved (Fig. 1A). The electron-density of LBs in conjunction with the fact that LBs are not limited by a classical trilaminar membrane (Fig. 1A) enables their unambiguous ultrastructural identification.

To characterize the LB two-dimensional ultrastructure in more detail, eosinophils freshly isolated from healthy donors were stimulated with different inflammatory stimuli (eotaxin-1/CCL-11), T cell expressed, and secreted (RANTES/CCL-5), tumor necrosis factor-alpha (TNF-α), interferon-gamma (IFN-γ) or medium alone for 1 h and prepared for conventional TEM. In addition, eosinophils from patients with the hypereosinophilic syndrome (HES) were studied. Some samples were also post-fixed with reduced osmium to increase contrast and facilitate identification of very small LBs. At least 450 electron micrographs randomly taken were carefully examined at different magnifications.

**Figure 2:**
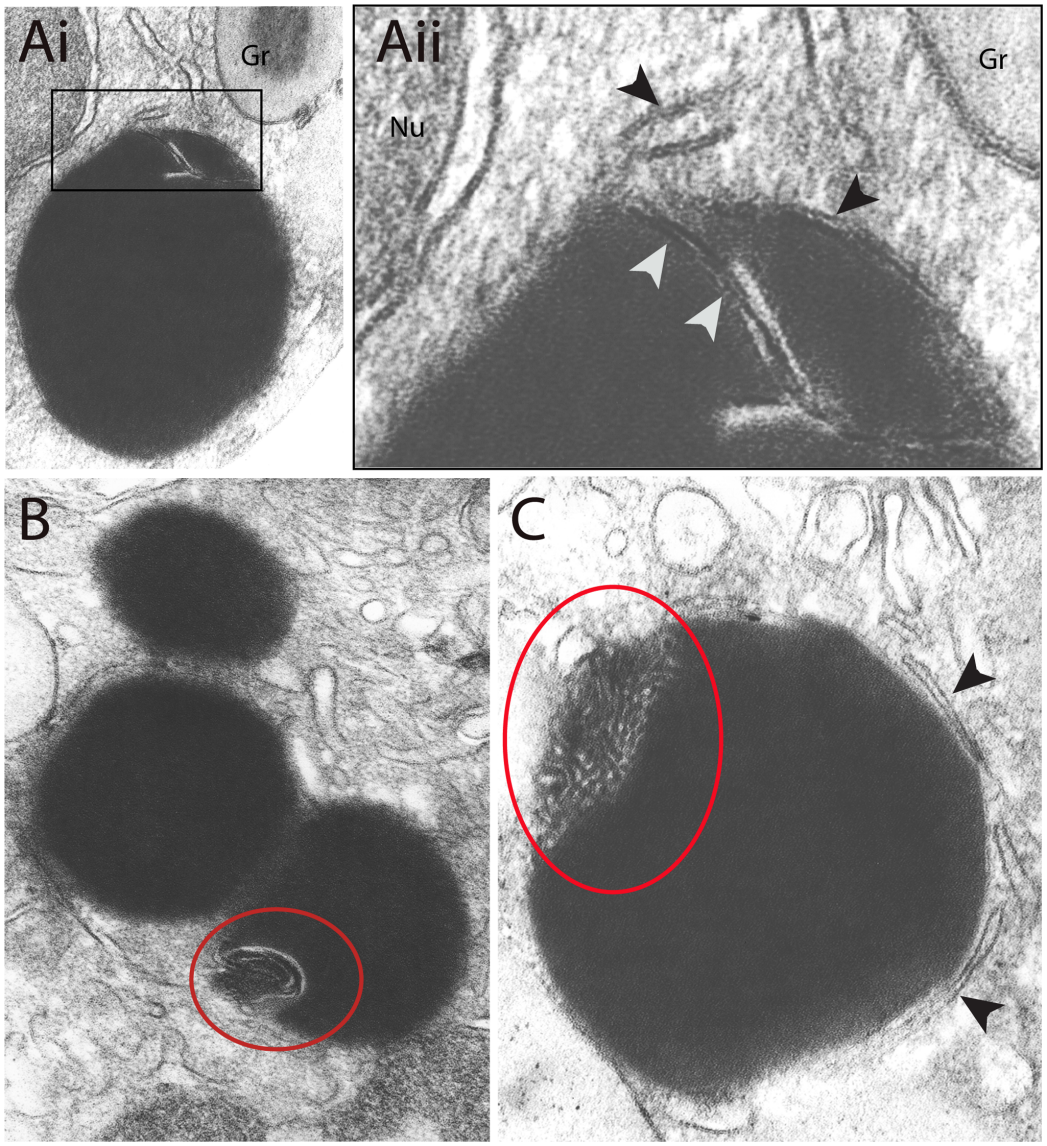
Two-dimensional ultrastructural aspects of lipid bodies (LBs) within human eosinophils. Conventional transmission electron microscopy (TEM) shows cytoplasmic LBs within unstimulated (Ai and Aii) and eotaxin-1 (B) or RANTES-stimulated eosinophils (C). In (Ai), a membranous structure (boxed area) is seen within a LB. Note in higher magnification (Aii) that the same trilaminar membrane aspect (white arrowheads) is also observed in surrounding cisternae of endoplasmic reticulum (ER) (black arrowheads) and around the nucleus (Nu) and specific granules (Gr). In (B) and (C) LBs show clear heterogeneous areas (circles) as part of their structure. Arrowheads in (C) indicate ER cisternae. Cells were stimulated or not with cytokines as before [22] and processed for TEM. Scale bar, 600 nm (Ai and B); 330 nm(Aii); 500 nm (C).

**Figure 3:**
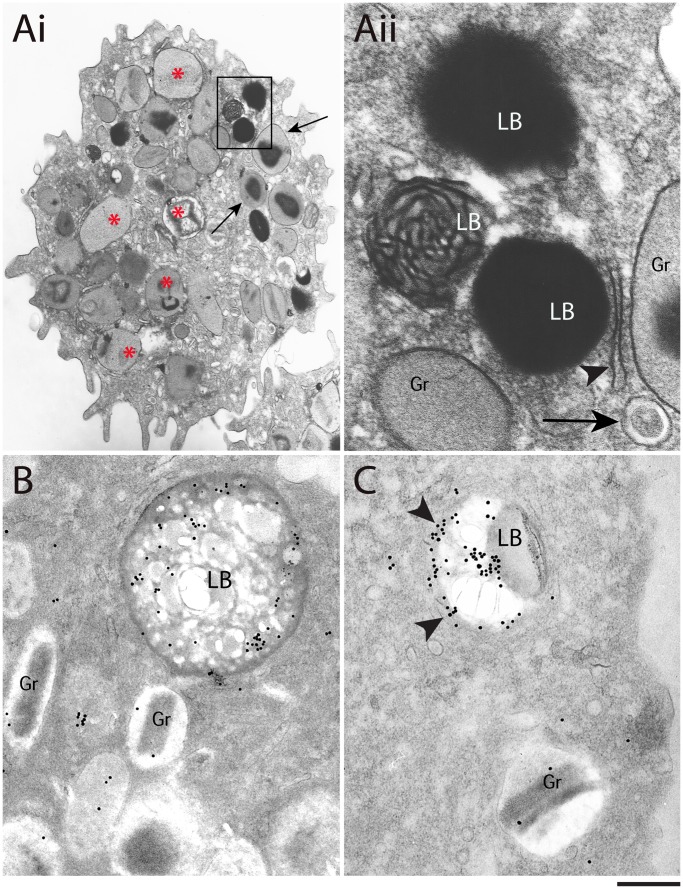
Lipid bodies (LBs) are not structurally homogeneous organelles. (A) A thin-section of an eotaxin-1-activated human eosinophil shows LBs (boxed area) and secretory granules in the cytoplasm. Note that most eosinophil granules (asterisks) exhibit morphologic features of piecemeal degranulation (reduced electron density and disarrangement of the crystaline core and matrix) as previously observed after stimulation with this chemokine [22]. Intact granules (arrows) with typical morphology are also observed. (Aii) Boxed area of Ai seen at higher magnification. The internal membranous structure of a LB can be clearly seen. An ER cisterna (arrowhead) and a typical eosinophil large transport vesicle (EoSV – eosinophil sombrero vesicle, arrow) are indicated. (B, C) Immunogold EM demonstrates intense cyclooxygenase (COX) labeling within LBs after an unmasking procedure which also revealed an internal heterogeneous area in (B). Note that COX labeling is observed in the LB core and periphery. In this case, labeling is probably associated with surrounding ER cisternae (arrowheads). Cells were stimulated with eotaxin-1 (100 ng/mL) for 1 h and processed for conventional TEM (A). For immunogold EM, cells from a hypereosinophilic donor were processed as in material and methods. Scale bar, nm 1 µm (Ai); 400 nm (Aii); 600 nm (B, C).

The numbers of LBs were quantitated in electron micrographs showing the entire cell profile and nucleus. LB numbers significantly increased in eosinophils stimulated with the 4 different cytokines compared to unstimulated controls (Fig. 1B). Greater LB formation was observed with the pro-inflammatory cytokines TNF-α and INF-γ compared with the stimulation with the two chemokines (eotaxin-1 and RANTES) (Fig. 1B and C). Hypereosinophilic eosinophils, which are naturally activated [28], also showed significantly greater numbers of LBs in the cytoplasm compared to unstimulated cells (Fig. 1B). In addition, activated cells showed a considerable variation in the LB sizes with very large LBs being observed in the cytoplasm in combination with small LBs (Fig. 1C). Although most LBs appeared homogenously electron-dense in conventional thin sections (∼80 nm thick) (Fig. 1A, C), heterogeneous densities (Fig. 1A, 2B–C, 3 B) and bilayer membranes showing the unambiguous trilaminar structure (Fig. 2A and 3A) were clearly identified within LBs under careful examination in all stimulated and unstimulated samples.

**Figure 4:**
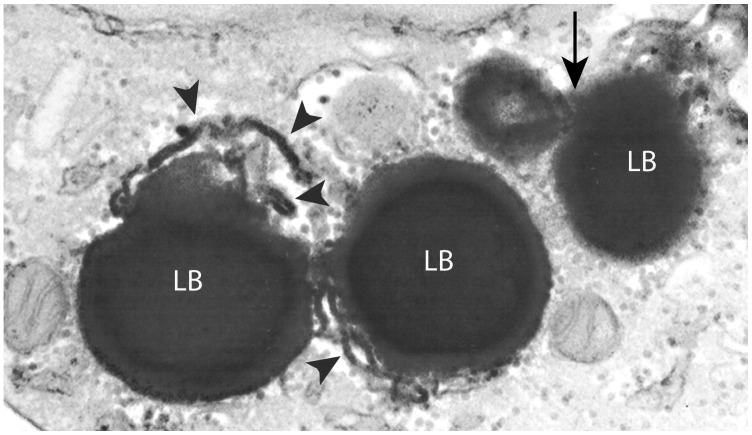
Lipid body (LB) association with the endoplasmic reticulum (ER). ER cisternae (arrowheads) are observed in close apposition to LBs and even partially intermingled in the lipid content of these organelles. Arrow indicates two fused LBs. Eosinophils were isolated from healthy donors, stimulated with tumor necrosis factor alpha (TNF–α) for 1 h before TEM processing with reduced osmium to increase contrast. Bar, 600 nm.

Next, the presence of the membrane-inserting protein cyclooxygenase (COX), also termed prostaglandin endoperoxide synthase, was investigated in naturally formed LBs from HES patients. COX expression was analyzed because this enzyme is linked to the synthesis of the inflammatory mediator prostaglandin E_2_ (PGE_2_), and is associated with intracellular membranes, including the ER [29]. Immunogold EM, preceded by a procedure to unmask LBs and restore protein antigenicity, revealed intense COX immunolabeling in LB cores (Fig. 3B, C) while the controls were negative. Labeling at the LB periphery, likely associated with the ER was also observed (Fig. 3C, arrowheads). In fact, a common characteristic of LBs was their association with the ER, which frequently appeared around (Fig. 2Aii, 2C, 3Aii) or even intermingled with the lipid content in the periphery of LBs in conventional thin sections (Fig. 2Aii). When samples were prepared with reduced osmium to increase contrast, ER cisternae in close association with LBs showed the same electron density as the LB content (Fig. 4, arrowheads).

### Electron Tomography Reveals a Complex Membranous System within LBs

**Figure 5:**
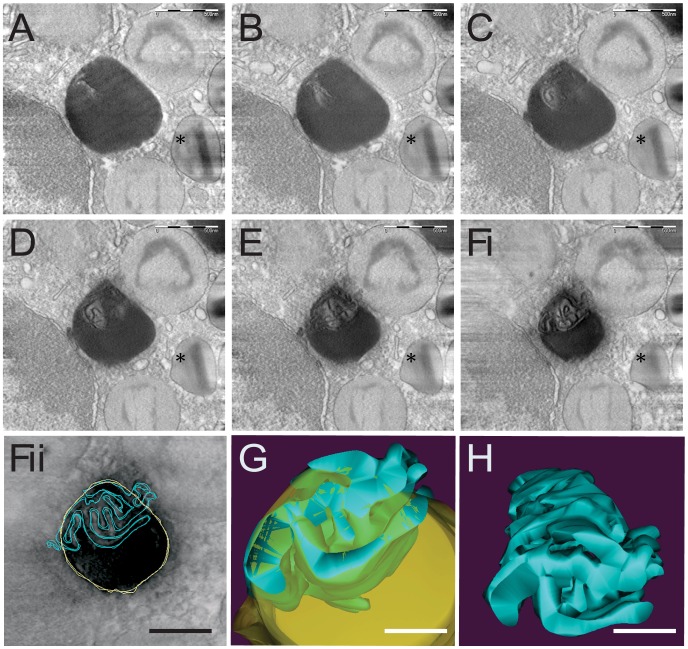
Electron tomography reveals membranes in the core of lipid bodies (LBs). (A–F) A gallery of 4 nm-digital slices of a tomogram taken from a representative human eosinophil stimulated with eotaxin-1. A typical eosinophil secretory granule (specific granule) is indicated (*). LB boundaries and internal membranes in consecutive tomogram slices were manually contoured in yellow and blue, respectively as in (Fii), and a computer-based 3D model is shown in (G) and (H). LB membranes are seen as interconnected tubules (H). Virtual slices were extracted from 3D reconstructions of a 400 nm eosinophil plastic section analyzed by fully automated electron tomography at 200 kV. Scale bar, 500 nm (A–Fi); 400 nm (Fii); 300 nm (G, H).

Although the analyses of thin sections showed occasionally the presence of membranes within LBs from both unstimulated (Fig. 2Aii) and stimulated eosinophils (Fig. 3Aii), a more accurate assessment required an analysis in 3D. To obtain information about the 3D structure of eosinophil LBs, we therefore undertook tomographic reconstructions of these cells. Electron tomography generates 3D structures of objects by combining multiple 2D images of the objects as they are systematically tilted along two orthogonal axes. The desired 3D structure is computed by back-projecting each 2D image with appropriate weighting [30]. We prepared 200–400 nm-thickness plastic sections from fixed cells for optimal LB preservation and contrast and analyzed at 200 Kv by fully automated dual-axis electron tomography. This approach enables the generation of a series of digital slices at different depths within the entire section thickness. Each digital slice is only a few nanometers thick (4 nm) and the structure and position of complex features can be determined by examining neighboring slices. Different virtual slices extracted from a representative tomogram are presented in Fig. 5A–F. In these slices, high resolution information about the internal organization of LBs is available. These slices showed that LBs, which generally appear as homogenous organelles in individual conventional EM thin sections (Fig. 1A, C), are actually composed of membranes within their core (Fig. 5A–F. See also Video S1). Interestingly, in serial sections showing less lipid content, from the same tomographic volume, these membranous structures were seen projecting to the outer surface of the LB boundary (Fig. 5Fi and Fii).

### Membranous Structures within LBs are Organized as Interconnected Tubules

**Figure 6:**
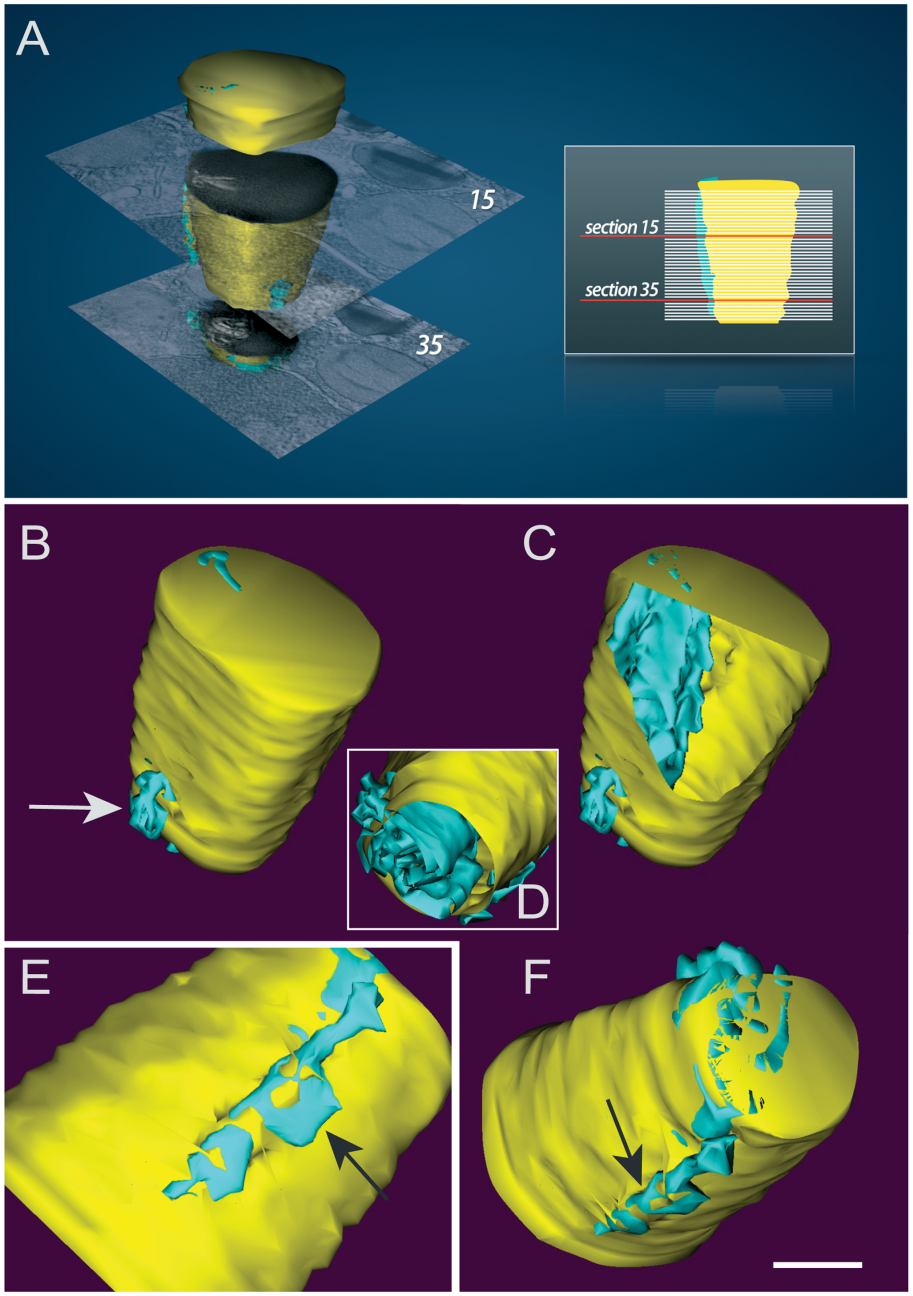
Different views of a lipid body (LB) 3D model. (A–F) Model constructed from hand-drawn contours marking the boundaries of the LB (yellow) and their associated membranes (blue) in a tomogram obtained from a human eosinophil. In (A), a composite to demonstrate 3D reconstruction from digital electron tomographic serial sections. Two sections are shown as part of a series from which a LB was reconstructed and rendered. Internal membranous structures are organized as an interconnected tubular system as seen in (C) and (D). Note the peripheral membranes (arrows) intermingled in the lipid content. Scale bar, 500 nm (B–F).

To gain insight into the 3D organization of LBs, features of interest in each section were abstracted as graphic objects to build a LB model (Fig. 5G–H and 6). We used a free editor (*Reconstruct*) for tracking both the LB and its membranous structures contours through the entire tomogram. By tracing these contours (Fig. 5Fii) on successive slices followed by 3D surfacing, an intriguing LB organization was revealed. The membranous structures appeared as complex interconnected tubules intermingled in the lipid content (Fig. 5G), resembling ER organization (Fig. 5H). The model was also revealing in demonstrating that membranous tubules seen as individualized structures in the periphery of LBs in some sections from the same tomographic volume blended together with the lipid content when all serial sections were combined (Fig. 6B–F, arrows). The likely presence of ER membranes in LBs revealed by electron tomography supports the previous demonstration of typical ER proteins within these organelles [10] and the observation of ER cisternae in close association to LBs as observed here (Fig. 2Aii, 2C, 3Aii, 4) and frequently reported in ultrastructural studies of LBs (reviewed in [31]).

## Discussion

### LB Formation within Activated Cells from the Immune System

A major advance in LB biology is the recognition of these organelles as inducible, newly formable organelles, elicitable in response to inflammatory stimuli. Different from cells associated with lipid storage and lipid-laden cell lineages that have a large number of cytoplasmic LDs, resting leukocytes have few LBs, but these cells can be rapidly stimulated to form new LBs (reviewed in [9]).

LB biogenesis is a process that happens *in vivo* during inflammatory reactions of different causes. Experimental and clinical inflammatory conditions such as infections with different pathogens including bacteria [5], [32]–[34], parasites [35]–[37] and viruses [38]–[39] as well as non-infectious diseases such as inflammatory arthritis [40], acute respiratory distress syndrome [41] and atherosclerotic lesions [42] induce intracellular LB accumulation in varied leukocyte types.

Here, leukocyte LB formation was evaluated, for the first time, by quantitativeTEM after stimulation with different stimuli. Our data showing increased, eotaxin-1-, RANTES-, TNF-α– or INF-γ –induced LB numbers within human eosinophils (Fig. 1B) support the fact that distinct signaling pathways can trigger LB formation within leukocytes. For instance, infection with *Mycobacterium bovis* BCG induces toll-like receptor 2 (TLR2)-mediated formation of LBs in macrophages [5] while the chemokines eotaxin-1 and RANTES, acting via CCR3 receptors, stimulate LB formation in eosinophils as demonstrated by fluorescence microscopy [43]. Moreover, we showed by TEM that naturally activated leukocytes, such as eosinophils from HES patients, have increased numbers of LBs in the cytoplasm.

Consistent with the role of leukocytes in inflammation, LBs formed by these cells constitute sites for the production of inflammatory lipid mediators (eicosanoids). LBs within inflammatory cells contain arachidonyl lipids, which serve as precursors for eicosanoid generation and all of the enzymes necessary for eicosanoid syntheses [4]–[5], [13], [44]–[45]. Therefore, the lipid and protein content of leukocyte LBs greatly differs from LDs found in other cell types. This differential content likely reflects in different organization of these lipid-rich organelles.

### LB Internal Membranes and Associated Proteins

Our comprehensive study of the LB ultrastructure by conventional TEM and using therefore 80 nm thickness-thin sections revealed occasionally the presence of membranes within these organelles (Fig. 2). These structures were found in LBs under different conditions including resting eosinophils (Fig. 2).

By using automated electron tomography, we identified that these structures are organized as a membranous system within LBs. One advantage of electron tomography is that it enables the generation of a series of just 4-nm-thick digital slices at different depths within the entire section thickness (Fig. 6A). Thus, it is possible to reveal, within the tomographic volume, structures that cannot be deduced from conventional 2D EM projection images in which they appear overlapped [30]. Another advantage is that electron tomography is the only technique that provides 3D information at very high resolution. The *z* axis resolution of the tomograms is 6–8 nm and the *x* and *y* axis resolution nearly 4 nm. Compared to imaging capabilities of other microscopic techniques, this z axis resolution is approximately 20-fold better than of classical EM thin sections and approximately 100-fold better than of typical images obtained with standard confocal light microscopic images (reviewed in [23]). Our results are based on both automated dual-axis acquisition and dual-axis tomogram reconstruction, which enables the generation of more accurate tomograms [24]. While our approach allowed the investigation of the LB architecture in its natural environment, it would also be interesting to evaluate the structure of purified LBs and their macromolecular complexes by electron tomography. However these studies are still hampered by the substantial fragility of these organelles during isolation and subsequent EM processing.

The identification of a membranous system within LBs may explain how proteins with transmembrane domains stay associated with the LB core in different cell types. In eosinophils and other immune cells, the presence of membrane proteins linked to the synthesis of inflammatory mediators, such as COX, 5-lipoxygenase (5-LO) and leukotriene C_4_ synthase (LTC_4_S), were localized fully within LBs as demonstrated here in human eosinophils (Fig. 3B, C) and in previous works using immunogold EM or fluorescence microscopy in eosinophils, neutrophils and macrophages [4]–[5], [13], [46]. Interestingly, a recent study demonstrated that stimulation of human eosinophils with the antimicrobial peptide LL-37 induced formation of LBs as well as assembly of 5-LO and transmembrane-spanning LTC_4_S within these organelles [45]. Likewise, the presence of membranes in the LB cores explains the presence of polar proteins such as TIP47 and adipose differentiation –related protein (ADRP/adipophilin/PLIN2) within LBs from adipocytes and macrophages [47].

LBs have been identified as sites of eicosanoid lipid mediator formation not only in leukocytes but also in other cells (e.g. human fetal membranes) [48]. Thus our insights into the internal architecture of leukocyte LBs is likely germane to other non-leukocyte cell types, contributing to the understanding of the regulated generation of eicosanoid intracrine and paracrine mediators. Moreover, our findings may be important to understand how LBs interact with membranous organelles and manage intracellular trafficking. For example, LBs within cells from the immune system consistently interact with phagosomes during infectious diseases, but little is known on how LBs exchange their content with phagosomes (reviewed in [49]). It is suggested that LBs act as platforms for managing the availability of proteins, functioning as transient sites for proteins that will be released, delivered or destructed [50].

### Implications to Understand LB Biogenesis

After computational alignment and reconstruction of the tilt series, followed by modeling, the 3D architecture of LBs could be appreciated. The organization of the LB internal membranes described here resembles that of the ER. Accordingly, our conventional TEM findings demonstrate portions of ER at the LB periphery and even intermingled in the lipid content. Although it is extensively recognized that lipid-rich organelles originate from the ER, it is still a matter of debate on how these organelles are formed. The prevailing model for LD biogenesis assumes that LDs are formed by accumulating neutral lipids between the cytoplasmic and luminal leaflets of ER bilayer membranes followed by the budding off of LDs surrounded by a phospholipid monolayer derived from the cytoplasmic leaflets of ER membranes (reviewed in [51]). This model is in agreement with the recognized phospholipid monolayer that surrounds LDs [14], but fails to elucidate a non-circumferential topology of membrane-associated proteins within LBs. By this model, proteins could insert only in the circumferential monolayer membrane of LDs or LBs, but, as noted, several proteins, including the perilipin family proteins which were initially described only at the LB periphery, are recognized as proteins incorporated within the LD cores [52]. In fact, little evidence supports the budding model and alternative models have been proposed (reviewed in [51]).

Our present findings using both electron tomography and conventional TEM support previous evidence for a new model of leukocyte LB formation by incorporating bilayer membranes of the ER within the LB cores [10]. This would explain the presence of ER transmembrane proteins such as calnexin and even ribosomes within LBs [10]. As the new model, LBs would be formed by incorporating multiple loops of ER membranes (both cytoplasmic and luminal leaflets of membranes) within developing LBs.

The growth of a lipid-rich organelle frequently involves the addition of large amounts of tryacylglycerols to its core. This means that triacylglycerol synthesis must be coordinated with the organelle growth. How newly synthesized triacylglycerol and other neutral lipids are delivered to the cores of nascent LBs has been unclear. Our model for LB biogenesis may explain the growth of LBs by accumulations of neutral lipids locally synthesized amongst the internal, ER-originated LB membranes. Indeed, several enzymes involved in the synthesis of triacylglycerol have been demonstrated in isolated LDs and associated with local synthesis of these lipids [53]–[54]. Moreover, the synthesis of lipids within LBs would elucidate the rapid and extraordinary enlargement of LBs in the cytoplasm of leukocytes during different diseases. Our group has been demonstrating LBs occupying large portions of the cytoplasm in response to clinical and experimental (Fig. 1C) inflammatory conditions, including infectious diseases (reviewed in [9], [49], [55]–[56]).

Hence, the recognition of ER-derived membranes within LBs may be crucial to understand the functional activities of LBs. We are just beginning to understand fundamental aspects of LB biogenesis and functions, and further studies are needed to determine the functional significance of the internal LB membranes, especially in the context of protein compartmentalization and management during cellular mechanisms of diseases.

### Leukocyte LBs versus Adipocyte LDs

The novel identification of organized internal membranes within leukocyte LBs provides a new concept of these organelles, differentiating LBs in structure and function from LDs. LDs from adipocytes are extensively described as organelles with neutral lipids in their cores and peripherally associated phopholipids and proteins, but leukocyte LBs do not share the same structure. Although a phospholipid hemi-membrane and proteins decorate the surface of leukocyte LBs, their cores are more complex locales.

Our present findings bring a new focus into the structure of lipid-rich organelles. We provide evidence that not only the function but also the spatial organization of these organelles may vary depending on the cell type. Therefore, analogous to the paradigm shift of LDs from inert lipid deposits to highly dynamic, multifunctional organelles, we propose a new view of LBs as organelles with incorporated membranes that are likely fundamental to LB functional capabilities such as managing of proteins and lipids and synthesis of eicosanoids in leukocytes.

## Materials and Methods

### Eosinophil Isolation and Viability

Granulocytes were isolated from the blood of different healthy and hypereosinophic donors. Eosinophils were enriched and purified by negative selection using human eosinophil enrichment cocktail (StemSep™, StemCell Technologies, Seattle WA) and the MACS bead procedure (Miltenyi Biotec, Auburn, CA), as described [57], with the exception that hypotonic red blood cell (RBC) lysis was omitted to avoid any potential for RBC lysis to affect eosinophil function. Experiments were approved by the Beth Israel Deaconess Medical Center Committee on Clinical Investigation, and informed consent was obtained from all subjects. Purified eosinophils (10^6^ cells/mL) from the healthy donors were stimulated with recombinant human eotaxin-1 (100 ng/mL; R&D Systems, Minneapolis, MN); RANTES (100 ng/mL; R & D Systems) TNF-α or INF-γ (200 ng/mL; R&D Systems) in RPMI-1640 medium plus 0.1% ovalbumin (OVA) (Sigma, St. Louis, MO), or medium alone at 37°C, for 1 h. Eosinophil viability and purity were greater than 99% as determined by ethidium bromide (Molecular Probes, Eugene, OR) incorporation and cytocentrifuged smears stained with HEMA 3 stain kit (Fisher Scientific, Houston, TX), respectively.

### Cell Preparation for TEM

Purified eosinophils were immediately fixed in suspension (not pelleted) in a mixture of freshly prepared aldehydes (1% paraformaldehyde and 1.25% glutaraldehyde) containing 2.5% CaCl_2_ in 0.1 M sodium cacodylate buffer, (pH 7.4) for 1 h, at room temperature (RT), washed in the same buffer and centrifuged at 1500 g for 1 min [10], [13]. Samples were then resuspended in molten 2% agar in 0.1M sodium cacodylate buffer, pH 7.4 and quickly recentrifuged. Agar pellets were post-fixed in 1% osmium tetroxide in sym-collidine buffer, pH 7.4, for 2 h at RT. After washing with sodium maleate buffer, pH 5.2, pellets were stained *en bloc* in 2% uranyl acetate in 0.05 M sodium maleate buffer, pH 6.0 for 2 h at RT and washed in the same buffer as before prior to dehydration in graded ethanols and infiltration and embedding with a propylene oxide-Epon sequence (Eponate 12 Resin; Ted Pella, Redding, CA). Alternatively, samples were post-fixed in 2% aqueous osmium tetroxide and 1.5% potassium ferrocyanide in 0.1 M sodium phosphate buffer, pH 6.0 (reduced osmium) before dehydration and embedding as above. After polymerization at 60°C for 16 h, thin sections were cut using a diamond knife on an ultramicrotome (Leica, Bannockburn, IL). Sections were mounted on uncoated 200-mesh copper grids (Ted Pella) before staining with lead citrate and viewed with a transmission electron microscope (CM 10; Philips, Eindhoven, The Netherlands) at 60 KV. At least 450 electron micrographs randomly taken at different magnifications were analyzed.

### Quantitative TEM Analysis of LBs

To quantify the number of LBs, a total of 295 electron micrographs showing the entire cell profile and nucleus were randomly taken from unstimulated and stimulated eosinophils and the numbers of LBs were counted. Data were compared by the Mann-Whitney “U” test (*P*<0.05).

### Immunogold Electron Microscopy

Freshly cut thin sections from eosinophils were collected on either gold or nickel grids and treated with 2% sodium methaperiodate for 30 min to unmask LBs and restore protein antigenicity [58]. Sections were then washed three times (10 min each) in Tris-buffered saline containing 0.1% bovine serum albumin (TBS-BSA buffer), blocked with TBS-BSA buffer containing 5% normal goat serum (NGS) for 1 h, and incubated with mouse monoclonal antibody IgG anti-COX (Cayman Chemical, MI, USA) (not characterized re specificities for COX-1 or COX-2) for 1 h at RT (1∶20 in TBS, 0.1% BSA, 1% NGS, 0.1 Tween 20) as before [13]. After incubation, sections were washed in TBS-BSA (3×10 min) and incubated with 20 nm gold-labeled goat anti-mouse IgG (EY Laboratories, CA, USA) at 1∶20 dilution in the same buffer as the primary antibody. After incubation with the secondary antibody, sections were washed in TBS-BSA buffer (5 min), followed by two washes in distilled water (5 min each), dried overnight and contrasted with lead citrate for 10 min. Two controls were performed: (i) primary antibody was replaced by an irrelevant antibody at the same dilution (IgG1, MOPC21; Cappel, PA, USA), and (ii) primary antibody was omitted. Specimens were examined as described for conventional TEM.

### Electron Tomography, 3D Reconstruction and Modeling

Epon sections of 200 or 400 nm were collected from chemically fixed eosinophils as above for analysis by electron tomography [22]. Tilt series were acquired fully automatically at 200 kV on a Tecnai Sphera microscope (FEI, Eidhoven, The Netherlands) using Xplore 3D software (FEI). Digital images of the structures of interest were recorded as they were tilted from - 65° to +65° at 1° intervals on a 1K Gatan 794 slow scan CCD camera. The tomograms were generated using Xplore 3D software (FEI). All tilted images were aligned to a common tilt axis using cross correlation and the volume was reconstructed by real space weighted back projection. Five tomograms were analyzed. Modeling was carried out using *Reconstruct* software [59].

## Supporting Information

Video S1 Electron tomography of a lipid body in the cytoplasm of a human blood eosinophil. Note the presence of internal membranous sub-compartments. Eosinophils were isolated from healthy donors, stimulated with 100 ng/mL eotaxin 1 for 1 h at 37°C, chemically fixed and embedded in Eponate. Thick sections (400 nm) were cut and analyzed by automated EM tomography (dual-axis, tilting from −65° to +65° at 1° intervals, at 200 kV). The tomogram corresponds to Fig. 5.
(AVI)
